# Fluctuating environmental adaptation shapes antibiotic survival in *Mycobacterium tuberculosis*

**DOI:** 10.71150/jm.2606004

**Published:** 2026-08-31

**Authors:** Eun Seon Chung

**Affiliations:** Department of Molecular Biology and Microbiology, Tufts University School of Medicine, Boston, MA 02111, USA

**Keywords:** *Mycobacterium tuberculosis*, antibiotic tolerance, fluctuating environments

## Abstract

*Mycobacterium tuberculosis* (Mtb) encounters diverse and fluctuating microenvironments during infection, including changes in nutrient availability and pH. While adaptation to individual host-associated conditions has been extensively studied, the impact of repeated environmental fluctuations on bacterial physiology and antibiotic survival remains unclear. In this study, we investigated how long-term adaptation to stable or fluctuating environments influences Mtb growth and drug responses. Mtb populations were serially passaged for six consecutive passages under combinations of different carbon sources and pH conditions that were either maintained consistently or altered across passages. Environmental history significantly affected bacterial growth dynamics and antibiotic survival. Notably, under identical final condition with cholesterol as a sole carbon source, populations adapted to a stable environment exhibited higher survival following bedaquiline and rifampicin treatment than populations exposed to fluctuating environments. These findings suggest that stable environments promote the optimization of growth and stress-response programs, whereas environmental fluctuations limit such optimization despite potentially increasing phenotypic heterogeneity and a possibility of survival. Together, our results identify environmental history as an important determinant of antibiotic survival in Mtb and highlight the need to consider dynamic host-like environments when investigating tuberculosis physiology and drug responses.

## Introduction

When *Mycobacterium tuberculosis* (Mtb), the causative agent of tuberculosis (TB), infects the host, immune cells surround the Mtb bacilli, forming granulomas. As granulomas develop, Mtb encounters dynamic and stressful environments, including acidic stress and nutrient shifts (Cadena et al., 2017; Chandra et al., 2022; Chung et al., 2022; Gouzy et al., 2021; Sarathy and Dartois, 2020; Vandal et al., 2009). For example, upon infecting macrophages, Mtb experiences mild to extreme acidic conditions that can eventually lead to growth arrest (Baker et al., 2019; Gouzy et al., 2021; Vandal et al., 2009). Additionally, when granulomas develop necrotic caseum from dying or foamy macrophages, the caseum contains host-derived lipids, including a high proportion of cholesterol (Agarwal et al., 2021; Guerrini et al., 2018; Krueger et al., 2025; Russell, 2007). Mtb can utilize this cholesterol as a carbon source inside the host to sustain survival. Together, these dynamic changes in pH and nutrients create an environment in which Mtb must continuously adapt, motivating our study of how Mtb responds to fluctuating host-relevant conditions.

Such adaptations align with the general ability of bacteria to adjust their bulk growth rate depending on the carbon source they are consuming (Andersent and von Meyenburgt, 1980; Monod, 1949; Towbin et al., 2017). Similarly, exposure to different environments, including variations in carbon sources and pH, drives Mtb bacilli to change their growth dynamics by altering metabolic pathways or entering growth arrest (Baker and Abramovitch, 2018; Gouzy et al., 2021; Lim et al., 2021; López-Agudelo et al., 2017). These adaptive changes in metabolism and growth under different environments can lead to variable drug responses, contributing to a prolonged therapy and frequent treatment failure (Samuels et al., 2022; Warner and Mizrahi, 2006). Therefore, studying how Mtb adapts to fluctuating environments is critical for understanding its drug responses in context and to help improve therapeutic outcomes.

Despite these insights, most studies have focused on uniform environmental conditions, and little is known about how fluctuating environmental conditions, which better mimic the dynamic microenvironments of host granulomas, affect Mtb growth, phenotypic heterogeneity, and drug response. Furthermore, the impact of cumulative environmental history, beyond immediate growth conditions, on Mtb growth and drug tolerance remains poorly understood.

In this study, we investigated how Mtb growth and drug response are shaped by fluctuating environments, specifically using acidic conditions and cholesterol as a carbon source, compared with stable environmental conditions. By measuring doubling time and survival under clinically relevant antibiotics across six sequential passages with different environmental combinations, we aimed to understand how environmental adaptation and history influence growth and drug tolerance in Mtb.

## Materials and Methods

### Bacterial strains

Two Mtb strains were used in this study: the CDC1551 strain and a clinical strain 24TB069, referred to here as isolate 1. The clinical strain was isolated from a TB patient in Vietnam.

### Bacterial culture and growth media

Mtb strains were grown in standard medium consisting of 7H9 broth (ThermoFisher; DF0713-17-9) supplemented with 0.05% Tween 80 (ThermoFisher; BP338-500), 0.2% glycerol (ThermoFisher; G33-1), and 10% Middlebrook OADC (ThermoFisher; B12351). Strains were initially grown from frozen stocks to an OD_600_ of 0.5–1.0 at 37°C with mild agitation (100 rpm). Cultures were then back-diluted to OD_600_ 0.05 and grown to mid-log phase (OD_600_ 0.5–1.0) before experimental use.

For growth passaging, four different growth media were used. The standard medium was adjusted to either pH 7.0 (S7.0) or pH 6.2 (S6.2) using MOPS or MES, respectively, and a base medium consisted of 7H9 supplemented with 0.5 g/L fatty acid-free BSA (ThermoFisher; BP9704100), 100 mM NaCl, 0.05% tyloxapol (Sigma; T8761-50G), and 0.2 mM cholesterol (Sigma; C3045-5G). The base medium with cholesterol was also adjusted to either pH 7.0 (C7.0) or pH 6.2 (C6.2).

### Growth passages for a fluctuating environment

For the first passage, cells grown to mid-log phase in standard unbuffered 7H9 medium were added to fresh conditioned medium and diluted to an OD_600_ of 0.05 for the cells in standard media and 0.4 for cells in cholesterol media. Due to the markedly different doubling times of Mtb in cholesterol media (~4 days) versus standard media (~1 day), cultures were inoculated at different initial OD values so that both reached comparable OD values (~0.8) after four days of incubation, allowing a fair comparison of physiological state and drug response at equivalent population densities. Each culture was incubated at 37°C for 4 days. This approach was used to match the final culture density at the time of transfer or antibiotic treatment, rather than the initial inoculum size. Comparing cells at more similar growth stages and physiological states allowed us to evaluate differences in drug survival more directly, while minimizing potential confounding effects from factors unrelated to the main purpose of this study. At the end of each passage, OD_600_ was measured, and doubling time was calculated as the time required for the bacterial culture (as indicated by OD_600_) to double. Cells were back-diluted to an OD_600_ of 0.05 (standard) or 0.4 (cholesterol) when transferred to the next passage using fresh conditioned medium. A total of eight different fluctuating environmental combinations were tested across 6 passages, consisting of S7.0, S6.2, C7.0, and C6.2.

### Colony-forming units assay (CFU assay)

At the end of the last passage, OD_600_ was measured, and each culture was diluted to OD_600_ 0.5 using fresh conditioned medium corresponding to its final passage condition. An aliquot of each OD_600_-normalized culture was serially diluted and plated, 10 µl/dilution, to determine the day 0 CFU immediately before antibiotic treatment. The remaining normalized culture was then seeded in 96-well plates (200 µl/well), and treated with bedaquiline, rifampicin, or pretomanid, each at 1 µg/ml. Because MIC values may vary among samples with different prior environmental histories, all samples were treated with a fixed high dose, at least five times higher than the previously reported MIC for the CDC1551 strain (Yang et al., 2019). Plates were incubated at 37°C in a standing incubator for 10 days. After treatment, cells were washed twice with 1× phosphate buffered saline with 0.2% Tween-80 (PBST), serially diluted, and plated on 7H10 agar. Plates were incubated at 37°C for approximately three weeks until colonies were visible. Survival rate was calculated by normalizing the CFU recovered after 10 days of drug treatment to the corresponding day 0 CFU measured from the same OD_600_-normalized culture before drug exposure: Survival rate = (CFU after 10 days of drug treatment)/(corresponding day 0 CFU before drug treatment) × 100 (%).

### Statistical analysis

Statistical analyses were conducted using GraphPad Prism version 10.6.1 (GraphPad Software, Inc.). Differences between groups were evaluated by a two-tailed, unpaired t test, and *p* < 0.05 was considered statistically significant. The six-passage environmental adaptation and drug treatment experiment was performed as a single biological replicate; therefore, growth and survival data were interpreted as observed group-level patterns rather than statistically tested comparisons.

## Results

### 6-Passage experimental design with combinations of 4 environment types

To expose the CDC1551 WT and clinical strain (isolate 1) to either stable or fluctuating environments, cells were subjected to six sequential passages under the same or different conditions to allow adaptation (Fig. 1). For the stable environment, the same growth condition was used across all six passages (Fig. 1, a–c), whereas for the fluctuating environment, different combinations of growth conditions were applied (Fig. 1, d–h). For growth conditions, four distinct media were used: standard 7H9 supplemented with OADC adjusted to either pH 7.0 (standard pH 7.0; S7.0) or pH 6.2 (standard pH 6.2; S6.2), or base media with cholesterol as the sole carbon source that is either adjusted to pH 7.0 (cholesterol pH 7.0; C7.0) or to pH 6.2 (cholesterol pH 6.2; C6.2).

C6.2 was initially tested as a stable, non-fluctuating control condition. However, under this combined cholesterol and acidic condition, Mtb growth became progressively slower over sequential passages, with substantial aggregation and clumping that prevented reliable growth measurements. Therefore, C6.2 was excluded from the stable condition analysis.

For each passage, the cultures were incubated for four days at 37°C to allow the bacteria to adapt. Eight different environmental combinations (Fig. 1, a–h) were used in this study, with the first three combinations (Fig. 1, a–c) representing consistent, non-fluctuating conditions.

### Environmental history influences Mtb growth dynamics and doubling time

When cells were grown in stable environments - passaging into the same condition throughout six passages (Fig. 1, a–c) - those cultured in cholesterol media exhibited the longest doubling times at the last passage, followed by pH 6.2 and pH 7.0 in standard media (Fig. 2) (for doubling time calculation, see ‘Materials and Methods’). After six passages, their doubling times were 128 h (C7.0, Fig. 2b, left), 21 h (S6.2, Fig. 2a, left), and 18 h (S7.0, Fig. 2c, left) in CDC1551 strain, and 256 h (C7.0, Fig. 2b, right), 30 h (S6.2, Fig. 2a, right), and 31 h (S7.0, Fig. 2c, right) in isolate 1.

Among the samples for which cholesterol media was the last environment, samples that experienced cholesterol consistently throughout all six passages had longer doubling times than those exposed to fluctuating conditions (Fig. 2, b vs. e and g). This difference may arise because cells exposed to standard media at intermediate passages grow faster due to readily metabolizable carbon sources, such as sugars (Atolia et al., 2020; Wang et al., 2019). As a result, residual metabolic activity and faster growth from these intermediate passages could carry over, effectively shortening the doubling time when the culture is returned to cholesterol media (C7.0). The observation suggests that the bacteria retain a physiological “memory” of prior environmental conditions, which is reflected in the doubling time and influences drug response. A doubling time is calculated from the beginning to the end of each passage.

### Final environment is critical for determining antibiotic tolerance

After six passages with different combinations of environmental conditions, the cells were treated with a high dose (1 µg/ml) of three TB antibiotics: bedaquiline, rifampicin, or pretomanid. The MIC values of the CDC1551 strain for these drugs are 0.2 µg/ml, 0.02 µg/ml, and 0.02 µg/ml, respectively (Yang et al., 2019). Precise MIC values for isolate 1 were not determined in this study. Because the MIC of samples may vary after exposure to different passages and environmental conditions, we chose to treat all samples with a single high dose to compare antibiotic survival across environmental conditions. We aimed to assess how effectively the drugs eradicate Mtb cells that have experienced different or fluctuating environmental conditions by measuring CFUs.

Before drug treatment, samples were normalized to OD_600_ 0.5 after the final passage and plated for day 0 CFU measurements (Fig. S1). When samples were grouped according to the final nutrient condition, standard medium versus cholesterol medium, the difference in CFU counts was not statistically significant, although cholesterol-grown samples exhibited a slight trend toward lower CFU counts (Fig. S1, right). These results suggest that OD_600_-based normalization broadly produced comparable CFU counts between standard- and cholesterol-grown samples, although minor nutrient- or strain-dependent differences in the OD_600_-to-CFU relationship cannot be fully excluded.

When the final (sixth) passage environment was cholesterol (C7.0), survival rates under bedaquiline and rifampicin were higher (Fig. 3, b, e, and g) than those of samples with standard media (S7.0 or S6.2) as a last environment. This pattern was consistent across both the CDC1551 strain and the isolate 1 clinical strain, indicating that nutrient composition - rich OADC versus cholesterol in this study - plays an important role in drug survival, with cholesterol as a sole carbon source contributing to higher drug tolerance, possibly mainly through slowed growth, with altered metabolic pathways potentially playing an additional role, as the activities of both bedaquiline and rifampicin are strongly influenced by the bacterial growth and metabolic state (Campbell et al., 2001; Dupont et al., 2017; Pawełczyk et al., 2021; Samuels et al., 2022; Zhu et al., 2023). However, for pretomanid, the pattern was reversed: cells with cholesterol as the final environment exhibited lower survival compared to cells that were grown in standard media possibly because pretomanid is active on non-growing cells as well as the growing cells (Mudde et al., 2022; Stancil et al., 2021). These observations suggest that the effect of environmental adaptation on drug survival varies depending on the antibiotic’s mechanism of actions. Because this experiment was performed as a single biological replicate, survival differences were interpreted as observed group-level patterns rather than statistically tested comparisons.

### Fluctuating environments reduce drug survival rates compared to stable environments in Mtb

When the final environmental condition was the same, cell cultures that experienced a consistent environment throughout all six passages exhibited higher antibiotic survival than cultures subjected to fluctuating environments (Fig. 3). For example, among samples b, e, and g, which all experienced C7.0 as the final environment, the consistently adapted sample (b) survived better than the two fluctuating samples (e and g), particularly in the CDC1551 strain (Fig. 3). A similar pattern was also observed in isolate 1 under bedaquiline and rifampicin treatment, although the magnitude of this difference was smaller than in CDC1551. These results suggest that adaptation to a stable environment may enable Mtb to develop a more optimized survival strategy against a specific antibiotic condition. By contrast, although fluctuating environments may promote phenotypic heterogeneity (bet-hedging) and generate subpopulations with increased stress tolerance, continual environmental shifts may simultaneously limit the full optimization of growth and stress-response programs required for maximal survival under a specific antibiotic condition.

## Discussion

In this study, we investigated how Mtb alters its growth and drug responses in fluctuating environments by comparing these characteristics between fluctuating and stable environments, using measurements of doubling time and survival under antibiotic treatment. Our results indicate that environmental adaptation in Mtb depends not only on the last environment but also on the cumulative experience of prior conditions. Mtb is known to employ diverse survival strategies in response to environmental changes and stress, including bimodal growth and bet-hedging (Bei et al., 2024; Chung et al., 2022; Parbhoo et al., 2022). Consequently, drug response is shaped by the surrounding environments experienced by the Mtb (Lee et al., 2019; Mishra et al., 2021; Parbhoo et al., 2022).

Samples adapted to stable environments achieved higher survival rates against specific antibiotics, whereas exposure to multiple environmental changes led to more complex drug tolerance patterns. Across all three antibiotics tested, when the last environment was identical, cells adapted to consistent environments exhibited higher survival rates than those exposed to fluctuating conditions, suggesting that stable adaptation enables a highly optimized survival strategy. However, this pattern was more evident in CDC1551 strain than in isolate 1. This strain-dependent difference may reflect variation in baseline drug susceptibility, growth physiology, cholesterol adaptation, metabolic state, or CFU recovery pattern after environmental adaptation (Culviner et al., 2025; Yoon et al., 2025). Therefore, the effect of environmental history on drug survival may depend not only on the environmental sequence itself but also on strain background.

Cells exposed to cholesterol as the final environment survived better under bedaquiline and rifampicin, but the pattern was reversed for pretomanid, where cells grown in standard media survived better. These differences align with each drug’s mechanism of action. Bedaquiline inhibits ATP synthase, depleting cellular ATP and causing growth inhibition (Dupont et al., 2017; Zhu et al., 2023), but cholesterol-adapted cells can enter a growth-arrested state, minimizing energy consumption and enhancing survival (Ouellet et al., 2011; Rodríguez et al., 2014; Soto-Ramirez et al., 2017). Rifampicin inhibits RNA polymerase, and growth-arrested cholesterol-adapted cells are less sensitive due to slowed or dormant-like growth (Campbell et al., 2001). In contrast, pretomanid, a nitroimidazole that generates reactive nitrogen species and damages DNA, proteins, and lipids, remains active against non-growing cells, and cholesterol-adapted cells may not fully activate detoxification pathways or nitroreductase enzymes, leading to lower survival (Mudde et al., 2022; Stancil et al., 2021).

The observation that cells exposed to fluctuating environments exhibit complex drug response patterns can be explained by increased phenotypic heterogeneity among individual cells, as reflected by variability in doubling times. Because Mtb experiences fluctuating environments inside the host, it is likely that Mtb exploits high phenotypic heterogeneity as a bet-hedging strategy, with each subpopulation exhibiting different levels of survivability to specific drugs. Consequently, even when multiple drugs are treated at the same time, as in current standard clinical regimens, it still remains difficult to completely eradicate the entire Mtb population.

Notably, differences in nutrient composition (cholesterol vs. rich nutrient) had a stronger impact on survival than the mild pH variations tested in this study (pH 6.2 vs. 7.0). However, this may reflect the use of only mildly acidic conditions. Future experiments with lower pH conditions will be necessary to fully assess the role of acidity in growth and drug response under fluctuating environments. Although cholesterol was used as the sole carbon source in this study to examine its effect on Mtb growth and drug survival, this condition represents a simplified experimental model rather than a direct mimic of the in vivo caseum environment. In vivo, Mtb is exposed to a complex mixture of host-derived nutrients, including cholesterol, fatty acids, and other carbon sources, particularly within necrotic caseous lesions (Agarwal et al., 2021; Guerrini et al., 2018; Krueger et al., 2025; Russell, 2007). Therefore, future studies using more physiologically relevant nutrient conditions will be important for understanding how host-like carbon environments shape Mtb adaptation and drug tolerance. Because the six-passage environmental adaptation experiment was performed as a single biological replicate, the observed group-level patterns should be validated in future experiments with biological replicates. Additionally, single-cell studies will be critical to reveal how phenotypic heterogeneity develops in response to environmental fluctuations and to identify which subpopulations preferentially survive antibiotic treatment with specific mechanisms of action.

## Figures and Tables

**Fig. 1.**
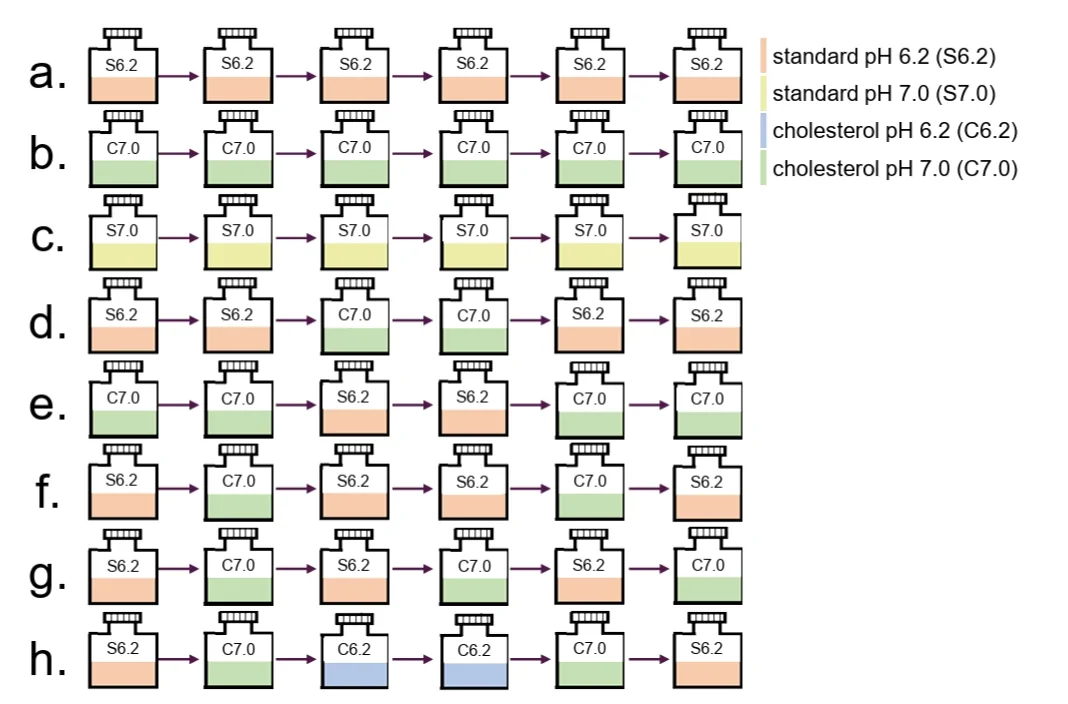
Schematic diagram of the fluctuating environment experimental design. Each row (a–h) represents a different combination of environmental conditions across six serial passages. Four environmental conditions were used: standard rich nutrient media at pH 6.2 (S6.2) and pH 7.0 (S7.0), and base media with cholesterol as a sole carbon source at pH 6.2 (C6.2) and pH 7.0 (C7.0). Each condition is color coded. Cells were cultured for four days for each passage.

**Fig. 2.**
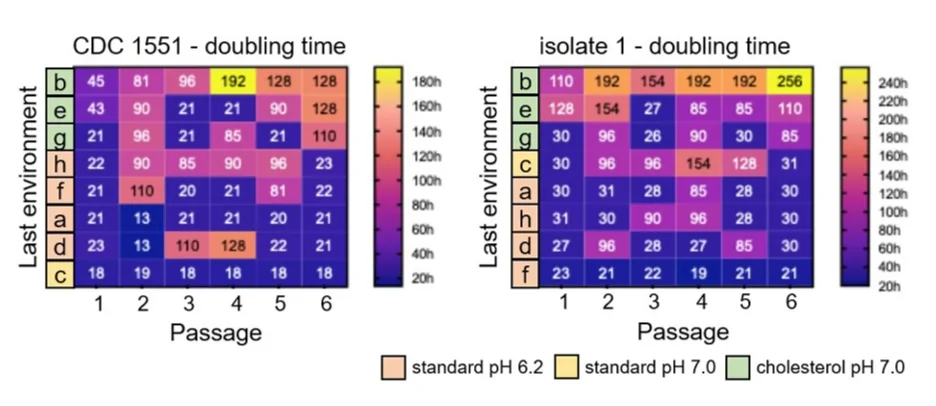
Doubling times of CDC1551 and isolate 1 strains at each passage. The rows represent different combinations of environmental conditions and are labeled from a to h, corresponding to the labels in Fig. 1. Each row is color-coded according to the last environment. Columns represent serial passages, with a total of six passages. The combinations are rank-ordered from the longest to the shortest doubling time at the last passage. The color bar indicates the doubling time.

**Fig. 3.**
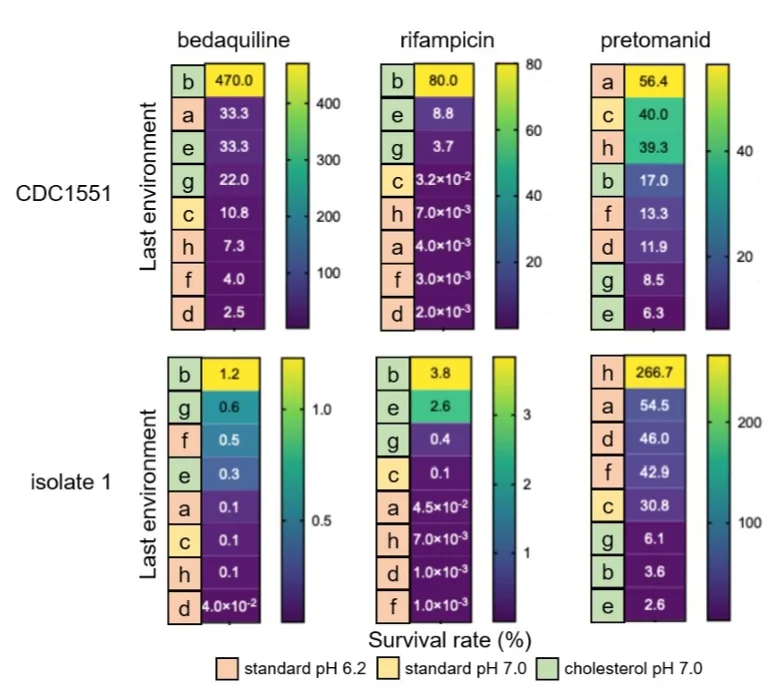
Antibiotic survival rates of CDC1551 and isolate 1 strains. Cells were treated with three drugs - bedaquiline, rifampicin, and pretomanid – at high dose (1 μg/ml) for 10 days each. Row labels correspond to different combinations of environmental conditions, as shown in Fig. 1. The combinations are rank-ordered from the highest to the lowest survival rate (top-to-bottom). Numbers in each row indicate the survival rate after drug treatment. The color bar represents the survival rate.

## Supplementary Information

Fig. S1. CFU counts after the final passage in standard- and cholesterol-grown samples. After the final passage, cultures of the CDC1551 strain and isolate 1 were diluted to OD600 0.5 and plated to determine CFU counts. CFU counts for each passage sample are shown on the left. For comparison, samples were grouped according to the final nutrient condition, standard medium, pH 6.2 or pH 7.0, versus cholesterol medium, pH 7.0 (right). The plots are presented with median values.
