# Supplementary material for: Fluctuating environmental adaptation shapes antibiotic survival in Mycobacterium tuberculosis
Source: J Microbiol. 2026 Aug 31;64(8):e2606004. doi: 10.71150/jm.2606004 (PMC13688124; doi:10.71150/jm.2606004)
Supplement: Supplementary file 1 [file jm-2606004-Supplementary-Fig-S1.pdf]

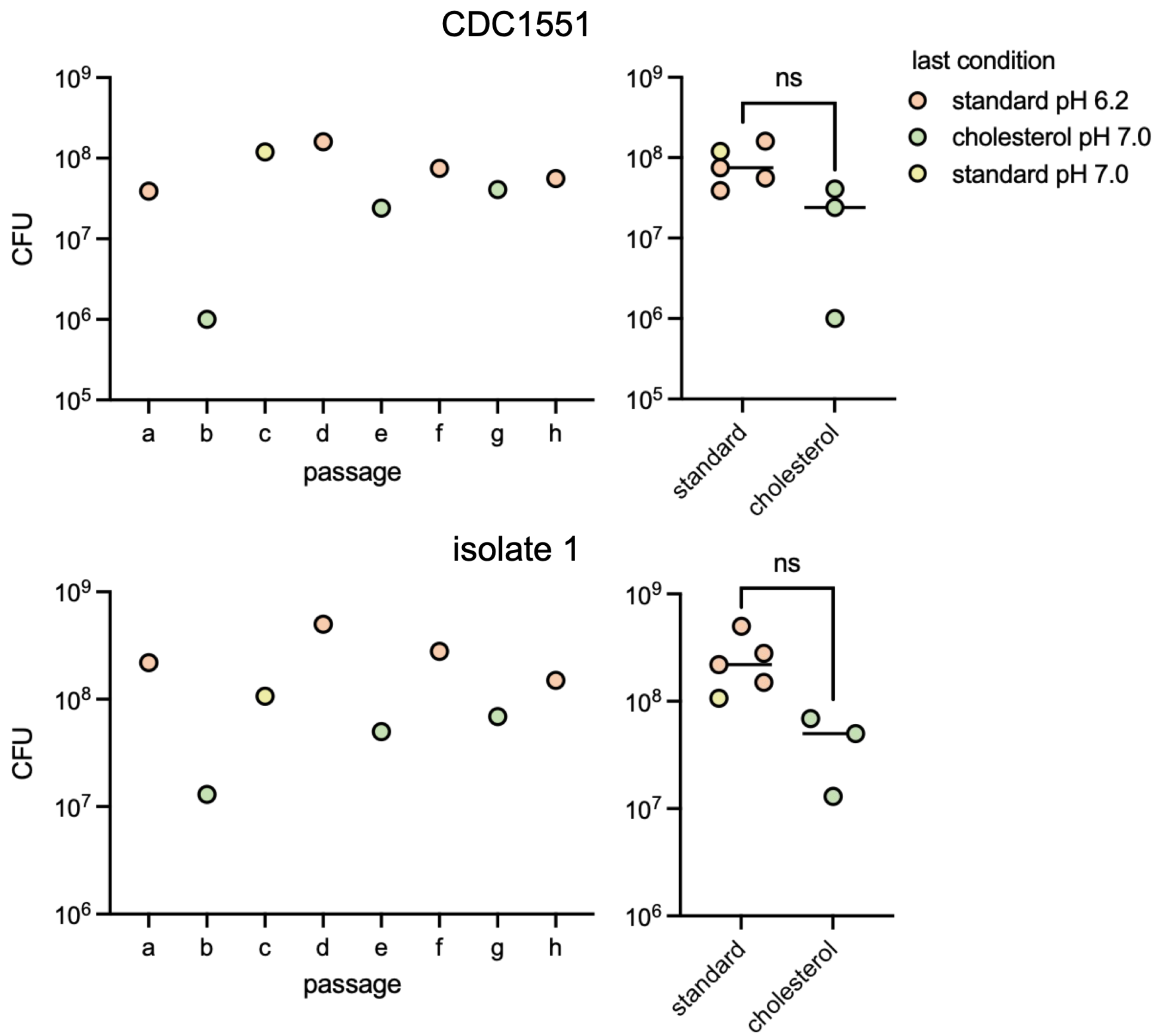

Fig. S1. CFU counts after the final passage in standard- and cholesterol-grown samples. After the final passage, cultures of the CDC1551 strain and isolate 1 were diluted to OD_{600} 0.5 and plated to determine CFU counts. CFU counts for each passage sample are shown on the left. For comparison, samples were grouped according to the final nutrient condition, standard medium, pH 6.2 or pH 7.0, versus cholesterol medium, pH 7.0 (right). The plots are presented with median values.
